# ICT Application and Use in Health Sciences Research at the Global Level: A Scientometric Study

**DOI:** 10.3390/healthcare10091701

**Published:** 2022-09-05

**Authors:** Naved Ahmad, Ibrahim Atoum, Jalaluddin Khan, Yazeed Alqahhas

**Affiliations:** 1 Department of Computer Science and Information Systems, College of Applied Sciences, AlMaarefa University, Riyadh 13731, Saudi Arabia; 2 Department of Computer Science and Engineering, Koneru Lakshmaiah Education Foundation, Guntur 520002, Andhra Pradesh, India

**Keywords:** ICT, health science, scientometric, citation, h-index

## Abstract

The growing use of information and communication technology has now expanded to health professionals in practice. This study aimed to highlight the current status of Information and Communication Technology (ICT) use in health sciences as reported in journal papers between 2002 and 2021. This paper presents the annual trends, top institutes and countries, citations, h-index, keywords distribution, and top authors in this research domain. The data were extracted from the Web of Science database, and R studio and Bibexcel tools were used for analysis. The study analyzed a total of 140 documents published over a span of two decades. Health Care Sciences Services (34) and Computer Science published the most health science articles (29). The USA (19) was the most productive country, followed by England (16) and the Netherlands (15). Berg M was the most productive author, with 36 articles. The results show that institutions such as Erasmus University and Duke University have published numerous articles on the topic, encouraged by specific R&D funding schemes, and made a significant contribution to the development of health sciences research. The findings of this study offer valuable information about international initiatives and projects relevant to the advancement of ICT in health science research, which may be utilized to pinpoint potential future study topics such as artificial intelligence development.

## 1. Introduction

Gustav Wagner (Germany) established the first professional association for health information in 1949 [1]. Health informatics, often known as health information systems, is a field that combines information sciences, computer science, and medicine. It is concerned with the resources, technologies, and procedures needed to optimize information acquisition, storage, retrieval, and use in health and biomedicine [2].

Health science is a broad term that encompasses several sub-disciplines that deal with the application of science in health [3]. Health sciences include traditional Western medicine and alternative medicine [4]. Humans have always had to cope with illness; therefore, health sciences have existed since the dawn of humanity. Medicine, nutrition, and other health-related topics and their effects on humans and animals are studied in this discipline (Shilpa et al., 2014).

Information and Communication Technology (ICT) is based on the development of digital technologies, databases, and other applications that aim to prevent illness, treat diseases, and manage chronic ailments in individuals and communities. They also provide the capacity for improving system efficiencies and preventing medical errors in health care delivery [5]. Health information technology allows for new and more efficient ways to access, communicate, process, and store data [6].

ICTs allow for remote care, and interdisciplinary clinical and knowledge support. These are all meant to bridge the gap between the health sector and other sectors in both developing and developed countries [7]. Since 2005, the World Health Organization has called on member states to develop “information and ICT infrastructure for health that is deemed appropriate to promote fair, affordable, and universal access to their facilities, and to use the information” and to “continue working with telecommunications companies and other partners to reduce costs and make e-health a success” [8]. Health and Education, Hospital Management Systems, Health Research and Health Data Management are four domains in which ICT is used in health sciences.

Scientometrics is the application of quantitative approaches to scientific communications to quantify the impact of research on society and compare its output and impact at the national and international levels [9]. These include assessing the influence of publications, journals and institutions/universities, deciphering the scientific citations, and mapping the research topics. There have been a variety of scientometric studies, many of which are based on the research output of countries and institutions. Several scientometric studies have been conducted with certain publications being selected and others, such as book reviews, editorial books, and conferences, being excluded.

This research is a scientometric analysis of the global research output on ICT usage in health sciences, published between 2002–2021. Publications were examined in terms of publication year, document categories, prolific authors, sources, institutions, and countries.

## 2. Review of Literature

**Sood and Rawat** highlighted the results of a scientometric examination of research conducted on disaster management employing ICT. For technical developments in ICT-assisted disaster management research, the growth of publications, citation analysis, collaborations, and keyword co-occurrence analysis was conducted. The results identified the lists of important publications, countries, and institutions that have made substantial contributions to this field of study. This study provided a foundation for future research on this topic by presenting the evidence of diverse patterns, research trends, and collaborations in the research domain [10].

**Vaquero-álvarez et al.,** conducted a bibliometric study in the healthcare sector. The major goal of the research was to focus on articles from the past 30 years on technology and workplace safety in the healthcare industry. The 1021 documents that were analyzed in the study demonstrated a growing trend by country, especially in the USA, and by year The analysis of journal co-citations found that major journals such as *Infection Control and Hospital Epidemiology* were linked to other important journals and played a key role in cluster formation [11].

**Zonneveld et al.,** conducted research on ICT in healthcare. They used databases such as MEDLINE, CINAHL, and the Cochrane Library for data collection. A total of eleven studies were identified. It was found that videoconferencing applications and the telephone were the most widely used technologies. In ten of the eleven studies, there was a change in participation in everyday life. Participation was primarily defined as being involved in everyday life circumstances or activities [12].

**Gaffar et al.,** evaluated publications on tourism indexed in the Web of Science from 2015 to 2019. A total of 16,941 numbers of records were retrieved for the study. The researchers analyzed various aspects of the publications such as year-based distribution of publications, document types, language-based distribution, country-based distribution, institution-based publications, and author-based publications based on the research objectives. The study also analyzed the highest and lowest records of the different aspects of the tourism literature published during the study period [13].

**Bm and Gupta** studied 1030 global publications on thalassemia research indexed in the Scopus database. The researchers investigated their growth rate, global share, quote effect, international collaborative document share, publication distribution by broad subject, productivity, top organizations, top authors’ citation profiles, preferred media of communication, and bibliographic characteristics of the highly cited papers [14].

**Krishnamoorthy and Amudhavalli** examined three decades of publications in health sciences published between 1970 and 2000 in India. The top three sub-disciplines identified were general medicine, pharmacology, and biochemistry. Among the top three sub-disciplines, organic chemistry led the way because of India’s R&D focus on organic chemistry with Artificial Intelligence (AI) 136, compared to general medicine (AI = 109) and pharmacology (AI = 87). However, the literature published on general medicine in India, at 6016publications, was significantly more than on biochemistry, with 1091 [15].

## 3. Objectives of the Study

This study aimed to analyze research on ICT use in health science. The main objectives of the research study were:

To find out the growth of publications with citations;

To determine the most productive authors;

To highlight the specific areas of research;

To determine which institutions were the most productive;

To identify the most prolific authors and their institutions.

## 4. Methodology

**Figure 1:**
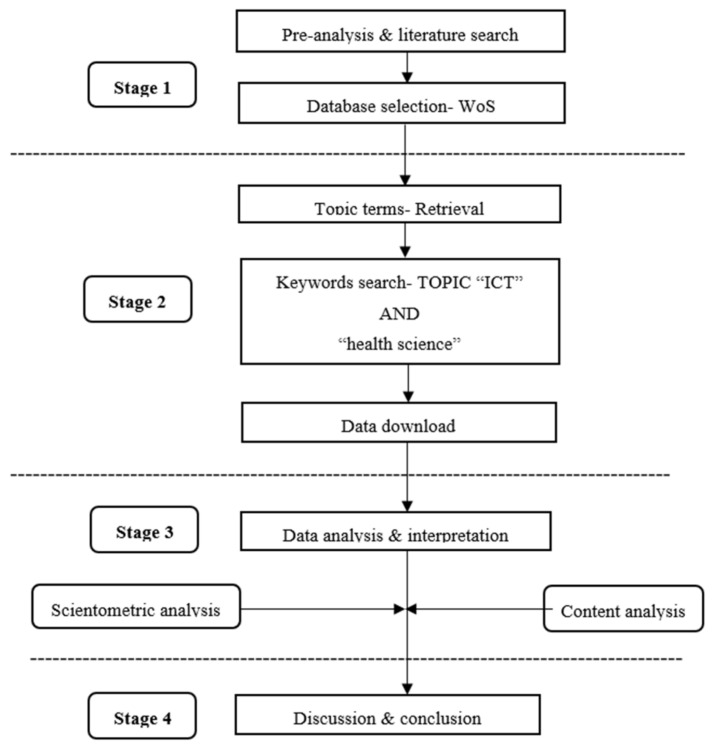
Research flowchart design.

The information was gathered using the Web of Science Database (Figure 1). Publications on the topic of ICT use in health science research published from around the world during the 2002 to 2021 period were retrieved. Various search strategies were developed and combined with the main search phrase to produce papers that could be used to evaluate data regarding countries, organizations, authors, sources, etc. [16]. The data were extracted from the WoS international database using the keywords “ICT” and “health science” for publications on ICT in health science research published from 2002 to 2021. A total of 140 records were downloaded and analyzed by using R Studio and Bibexcel software based on the study objectives.

## 5. Analysis and Interpretation

We used the concept of scientometric study as a technique that analyses scientific databases to infer links between citations to academic journals, and to identify patterns and future paths for research on particular themes. Through a methodical examination of authors and journal citation records, the idea aids in the better understanding of the various elements of science. Therefore, the goal of this scientometric analysis is to evaluate the status and trends of ICT in health science research from the last two decades. The findings of this research may be used to examine the features of health science publications, in addition to giving an overview of health science publications based on predetermined criteria (such as nations/regions, organisations, prolific authors, and journals, among others).

### 5.1. Annual Growth of Publications with the Number of Citations

**Figure 2:**
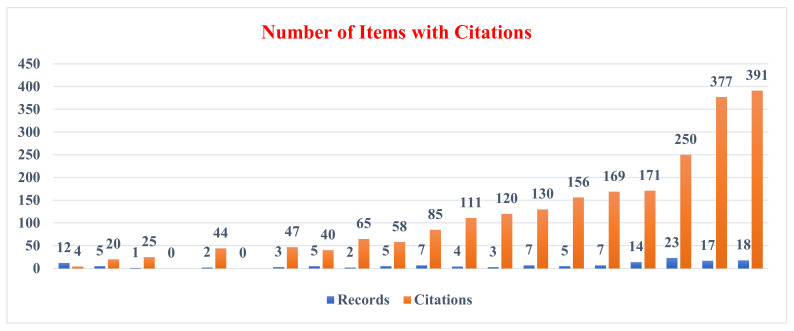
Number of records with citations in health sciences research.

Table 1 and Figure 2 display the year-by-year publishing pattern in health sciences research around the world between 2002 and 2021. There were 23 papers published in the year 2019, which was a significant increase from previous years. There were 12 documents published in 2002, followed by a decrease in activity in the ICT in health sciences research between 2003 and 2008. There were no publications in the year 2005 and the year 2007. The period between 2018 and 2021 finally saw a considerable number of publications being published on health science research, totaling 72 records. These 140 records received a total of 22,663 citations, with more than 100 yearly citations received in the 2013–2021 period.

### 5.2. Prolific Authors

Between 2002 and 2021, the top 10 most productive authors out of the 590 authors who were active in health sciences research, published 140 records. Table 2 shows the scientometric characteristics of these 10 authors, as well as their research output, citations, and h-index values. Nine authors published two documents, except for Berg M, who published three documents with the highest three h-indexes received. Five authors had the highest number of citations (four) received among the top ten authors.

### 5.3. Language-Wise Distributions

**Figure 3:**
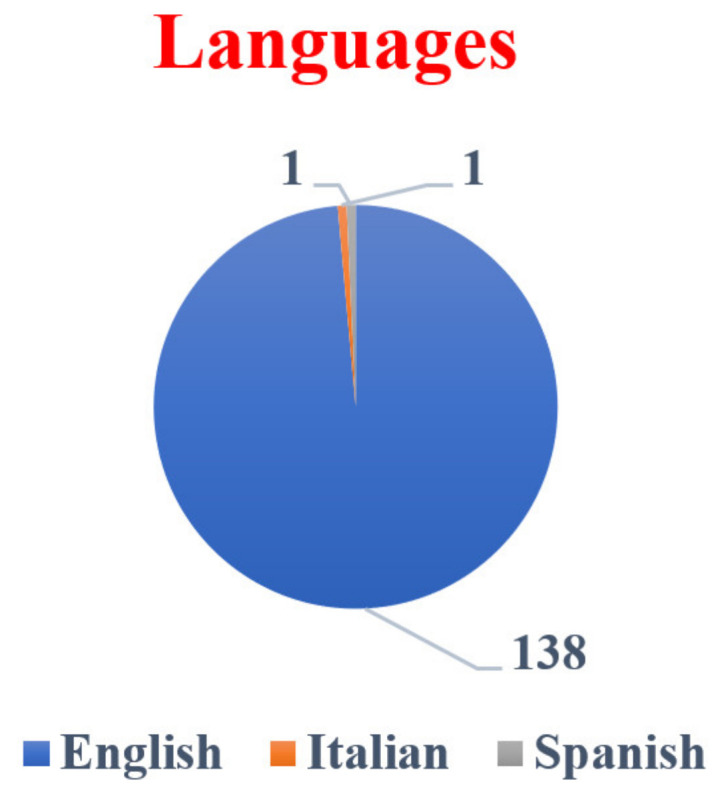
Language-wise distributions in health sciences research.

Figure 3 depicts how scientists working in the field of health sciences research around the world liked to publish their findings in their respective fields. The majority of the articles (138 or 98.57%) were in the English language, with one (0.71%) article each in Italian and Spanish.

### 5.4. Document Types

Table 3 shows the distribution of publications on the subject of ICT usage in health sciences by document type. Between 2002 and 2021, a total of 140 papers on health sciences research were published. The vast majority of the papers (106, 75.71%) appeared as journal articles and had the highest number of citations (1463) received with an h-index of 19, followed by review articles (20, 14.28%) with 456 citations, proceedings paper (6, 4.28%) with 41 citations, editorial materials (5, 3.57%) with 30 citations, and then early access (2, 1.42%), and finally book reviews (1, 0.71%). On the other hand, early access and book reviews did not receive any citations or h-index, and only one book review was published in 2012.

### 5.5. Research Area Distributions

**Figure 4:**
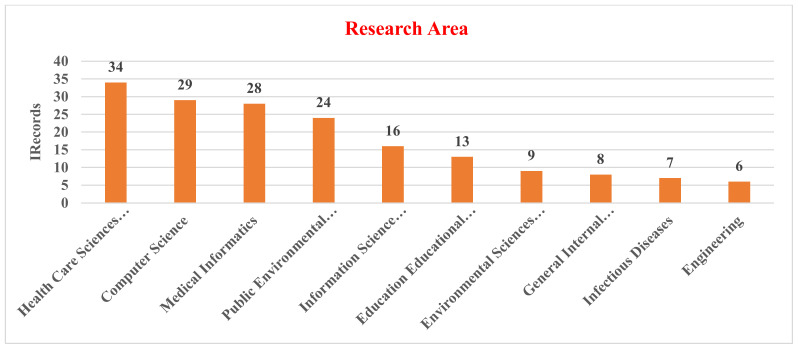
Research area distributions in health science research.

Research area analysis of the documents provided by the Web of Science database was conducted. Figure 4 provides the results of an analysis of the distribution of publications in health sciences research based on broad research area categories. The figure indicates that ICT use in health sciences research has been published in several broad research areas. The highest publication output came from the Health Care Sciences Services (34, 24.29% documents). This indicates that most of the research has been conducted in the field of Health Care Sciences Services, followed by Computer Science (29, 20.71% documents), Medical Informatics (28, 20.00% documents), Public Environmental Occupational Health (24, 17.14% documents), and finally Information Science Library Science (16, 11.43% documents).

### 5.6. Distributions of Publishers

The global publication output in health sciences research in the context of different publishers was analyzed for the 140 documents (Table 4). Elsevier (40, 28.57% papers) led the way with the highest number of publications, followed by Springer Nature (23, 16.43% papers), MDPI, and Taylor & Francis (11, 7.86% papers). Elsevier also had the highest number of citations (663) and an h-index of 15. The publisher Sage had four citations in one year in 2019.

### 5.7. International Collaboration

Table 5 shows how authors from around the world collaborated on health-related research publications. The selected documents were evaluated to determine the nature of the international collaborations among countries. According to the data, the United States of America led the way with the most articles (19, 13.57%) published collaboratively on health sciences research globally. England was ranked second on the list with 16 collaboratively published articles, followed by the Netherlands with 15 such records and the highest number of citations (508) among all. India was ranked sixth on the list of top 10 collaborators, with 11 collaboratively published articles receiving 41 citations.

### 5.8. Active Organizations

Table 6 shows the top ten most productive organizations, as well as their total global publications and total citations from 2002 to 2021. The table shows that the top ten most prolific health sciences research institutions have published two or more publications during the period and contributed to global research. These top ten organizations/universities had contributed a total of 26 articles published through international collaborations. Erasmus University published the most documents (5), followed by Duke University (2), Leiden University (2), and Makerere University (2). Furthermore, Erasmus University received the highest number of citations (293).

### 5.9. Important Keywords

Table 7 indicates the keywords used in ICT usage in health sciences research in the worldwide publications on the topic during the 2002–2021 period. A total of 140 documents were found with 1022 keywords. The most used keyword was identified as ‘ICT’ (22, 2.15%), followed by ‘care’ (18, 1.76%), and ‘health’ (14, 1.37%). This shows that the majority of research has taken place on the subject of the use of ICT in health sciences (ICT).

## 6. Discussion

This study conducted a scientometric examination of health-related articles published during the 2002 to 2021 period, and uncovered several intriguing findings regarding the usage of ICT in health-related publications. It explored the global publishing trend seen in 140 research papers on ICTs in health science published between 2002 to 2021 and indexed in the WoS database. Although the trend of publishing has risen in tandem with the advancement of ICT, this trend appears to have accelerated significantly after 2002, with some decreased activity in the middle of the study period. The growth in the number of publications then increased between 2019 and 2021. Interestingly, the total number of health science publications remained at zero during the years 2005 and 2007. The findings also revealed that the majority of the publications were emerging from the United States of America and other industrialized countries, with the USA contributing the most publications. The USA also had the most collaborative articles, followed by England and the Netherlands. In terms of institutes, Erasmus University had the most publications with the largest number of citations, followed by Duke University. According to the findings, the patient-entered paradigm will be an unavoidable trend in future medical progress via ICT.

Our study demonstrated how the use of bibliometrics in medicine transitioned into routine clinical activities, and as a result, our study may serve to advance the use of bibliometrics in healthcare. The study’s findings could also be utilized to teach medical students how to avoid common mistakes, learn about scientific discoveries and advancements, and develop new critical thinking skills about the use of bibliometrics in medical practice.

This study’s strength comes from its historical bibliometrics analysis of bibliometrics’ use in health science. The investigation was restricted to Web of Science-indexed papers, so choosing a different bibliographic database would produce somewhat different results. However, because the authors’ thematic analysis was qualitative and hence subjective, other researchers’ analyses might have produced alternative themes and therapeutic topics.

## 7. Conclusions

In conclusion, the current analysis identified a good trend in the development of literature for bibliometric analysis in health science. Our study also demonstrated that, in terms of general bibliometrics knowledge development, health science was at the forefront. For health care researchers and professionals, bibliometrics holds great promise as a source of fresh data on scholarly trends, medication, disease, and other developments in the health sciences.

## Figures and Tables

**Table 1:** Growth of publications and citations of the world output in health sciences research.

Year	Records	% of Records	Cumulative	% of Cumulative	Citations
2002	12	8.57	00	00	4
2003	5	3.57	17	1.73	20
2004	1	0.71	18	1.84	25
2005	0	0.00	18	1.84	--
2006	2	1.43	20	2.04	44
2007	0	0.00	20	2.04	--
2008	3	2.14	23	2.35	47
2009	5	3.57	28	2.86	40
2010	2	1.43	30	3.06	65
2011	5	3.57	35	3.57	58
2012	7	5.00	42	4.29	85
2013	4	2.86	46	4.69	111
2014	3	2.14	49	5.00	120
2015	7	5.00	56	5.71	130
2016	5	3.57	61	6.22	156
2017	7	5.00	68	6.94	169
2018	14	10.00	82	8.37	171
2019	23	16.43	105	10.71	250
2020	17	12.14	122	12.45	377
2021	18	12.86	140	14.29	391
	140	100		100	

**Table 2:** The contribution and influence of highly productive authors in health sciences research.

S. No.	Authors	Records	%	Citations	h-Index
1	Berg M	3	2.14	3	3
2	Abdullah AS	2	1.43	4	2
3	Agarwal R	2	1.43	2	2
4	Friedman RH	2	1.43	4	2
5	Grant A	2	1.43	0	2
6	Guillen-Gamez FD	2	1.43	2	1
7	Haux R	2	1.43	3	2
8	He HM	2	1.43	4	2
9	Huang KY	2	1.43	4	2
10	Ma ZY	2	1.43	4	2

**Table 3:** Document types in health sciences research.

Documents	Records	%	Citations	h-Index
Articles	106	75.71	1463	19
Review Articles	20	14.28	456	10
Proceedings Paper	6	4.28	41	5
Editorial Materials	5	3.57	30	3
Early Access	2	1.42	0	0
Book Reviews	1	0.71	0	0
**Total**	**140**	**100**		

**Table 4:** Distributions of publishers in health science research.

S. No.	Publishers	Records	%	Citations	h-Index
1	Elsevier	40	28.57	663	15
2	Springer Nature	23	16.43	224	7
3	MDPI	11	7.86	56	4
4	Taylor & Francis	11	7.86	133	5
5	Wiley	8	5.71	86	5
6	JMIR Publications, Inc	4	2.86	58	3
7	BMJ Publishing Group	3	2.14	5	3
8	Oxford Univ. Press	3	2.14	10	2
9	Sage	3	2.14	4	2
10	Thieme Medical Publishers	3	2.14	4	3

**Table 5:** The number and proportion of international collaboration articles published by the top ten most productive countries in health sciences research.

S. No.	Country	Records	%	Citations
1	USA	19	13.57	305
2	England	16	11.43	413
3	Netherlands	15	10.71	508
4	Canada	12	8.57	114
5	China	12	8.57	262
6	India	11	7.86	41
7	Germany	10	7.14	182
8	Spain	10	7.14	198
9	South Korea	6	4.29	19
10	Australia	4	2.86	227

**Table 6:** Number and share of international collaboration articles of organizations produced by the top ten most productive universities in health sciences research.

S. No.	Organization	Records	%	Citations
1	Erasmus University	5	3.57	293
2	Duke University	3	2.14	8
3	Leiden University	3	2.14	96
4	Makerere University	3	2.14	20
5	Boston University	2	1.43	8
6	De Montfort University	2	1.43	11
7	Duke Kunshan Univ	2	1.43	8
8	Eindhoven University Technol	2	1.43	26
9	Fudan University	2	1.43	17
10	Guangxi Med University	2	1.43	8

**Table 7:** Health sciences publications by top ten important keywords.

S. No.	Keyword	Occurrences	%
1	ICT	22	2.15
2	Care	18	1.76
3	Health	14	1.37
4	Internet	12	1.17
5	Technology	12	1.17
6	Education	11	1.08
7	Information	11	1.08
8	Science	11	1.08
9	Telemedicine	11	1.08
10	Challenges	9	0.88

## Data Availability

Not applicable.

## References

[1] Kulikowski C.A. (2017). Historical Roots of International Biomedical and Health Informatics: The Road to IFIP-TC4 and IMIA through Cybernetic Medicine and the Elsinore Meetings. Yearb. Med. Inform..

[2] Deserno T.M., Dugas M., Löbe M., Stausberg J. (2021). A Topical Collection on ICT for Health Science Research–EFMI Special Topic Conference. J. Med. Syst..

[3] Westbrook J.I., Braithwaite J., Gibson K., Paoloni R., Callen J., Georgiou A. (2009). Use of information and communication technologies to support effective work practice innovation in the health sector: A multi-site study. BMC Health Ser. Res..

[4] Shilpa G., Mathias J., Babu L., Abraham L., Mathew L., George L. (2014). Effectiveness of teaching programme on knowledge of mothers regarding the effect of family conflicts on school children. Arch. Med. Health Sci..

[5] Meetoo D., Rylance R., Abuhaimid H.A. (2018). Health care in a technological world. Br. J. Nurs..

[6] Iqbal S., Tariq M., Ayesha H., Ayesha N. (2021). AI Technologies in Health-care Applications. Artificial Intelligence and Internet of Things.

[7] Arya V., Deshmukh S., Bhatnagar N. (2015). High Technology Health Care Supply Chains: Issues in Collaboration. Procedia-Soc. Behav. Sci..

[8] Brenner M., Larkin P.J., Hilliard C., Cawley D., Howlin F., Connolly M. (2015). Parents’ perspectives of the transition to home when a child has complex technological health care needs. Int. J. Integr. Care.

[9] Theodore D.D., Shree S., Reddy A.A., Kuriokose R. (2016). Synergy: Information technology and health sciences. Arch. Med. Health Sci..

[10] Sood S.K., Rawat K.S. (2021). A scientometric analysis of ICT-assisted disaster management. Nat. Hazards.

[11] Bm G., Gupta R. (2018). Global Thalassemia Research: A Scientometric Assessment of Publications Output during 2008–17. Open Acc. Blood Res. Trans. J..

[12] Krishnamoorthy G., Amudhavalli A. (2008). Health Sciences in India: A Scientometric Study. Collnet. J. Sci. Inf. Manag..

[13] Vaquero-Álvarez E., Cubero-Atienza A., Ruiz-Martínez P., Vaquero-Abellán M., Mecías M.D.R., Aparicio-Martínez P. (2020). Bibliometric Study of Technology and Occupational Health in Healthcare Sector: A Worldwide Trend to the Future. Int. J. Environ. Res. Public Health.

[14] Zonneveld M., Patomella A.-H., Asaba E., Guidetti S. (2020). The use of information and communication technology in healthcare to improve participation in everyday life: A scoping review. Disabil. Rehabil..

[15] Gaffar S.A., Kumar S.K., Hossain S. Research Productivity of Tourism Literature (Global Level): A Scientometric Analysis. https://digitalcommons.unl.edu/libphilprac/4107/.

[16] Amees M., Hossain S., Batch M.S. 20 Years of Dentistry Research at World Perspectives: A Scientometric Study. https://digitalcommons.unl.edu/libphilprac/4450/.

